# Enhancement of hepatitis virus immunoassay outcome predictions in imbalanced routine pathology data by data balancing and feature selection before the application of support vector machines

**DOI:** 10.1186/s12911-017-0522-5

**Published:** 2017-08-14

**Authors:** Alice M. Richardson, Brett A. Lidbury

**Affiliations:** 1 Present address: National Centre for Epidemiology & Population Health, Australian National University, Canberra, ACT 2601 Australia; 2 Pattern Recognition & Pathology, Department of Genome Sciences, The John Curtin School of Medical Research, Australian National University, Canberra, ACT 2601 Australia

**Keywords:** Analysis of variance, Hepatitis B, Hepatitis C, Machine learning, Random forests, Synthetic minority oversampling technique

## Abstract

**Background:**

Data mining techniques such as support vector machines (SVMs) have been successfully used to predict outcomes for complex problems, including for human health. Much health data is imbalanced, with many more controls than positive cases.

**Methods:**

The impact of three balancing methods and one feature selection method is explored, to assess the ability of SVMs to classify imbalanced diagnostic pathology data associated with the laboratory diagnosis of hepatitis B (HBV) and hepatitis C (HCV) infections. Random forests (RFs) for predictor variable selection, and data reshaping to overcome a large imbalance of negative to positive test results in relation to HBV and HCV immunoassay results, are examined. The methodology is illustrated using data from ACT Pathology (Canberra, Australia), consisting of laboratory test records from 18,625 individuals who underwent hepatitis virus testing over the decade from 1997 to 2007.

**Results:**

Overall, the prediction of HCV test results by immunoassay was more accurate than for HBV immunoassay results associated with identical routine pathology predictor variable data. HBV and HCV negative results were vastly in excess of positive results, so three approaches to handling the negative/positive data imbalance were compared. Generating datasets by the Synthetic Minority Oversampling Technique (SMOTE) resulted in significantly more accurate prediction than single downsizing or multiple downsizing (MDS) of the dataset. For downsized data sets, applying a RF for predictor variable selection had a small effect on the performance, which varied depending on the virus. For SMOTE, a RF had a negative effect on performance. An analysis of variance of the performance across settings supports these findings. Finally, age and assay results for alanine aminotransferase (ALT), sodium for HBV and urea for HCV were found to have a significant impact upon laboratory diagnosis of HBV or HCV infection using an optimised SVM model.

**Conclusions:**

Laboratories looking to include machine learning via SVM as part of their decision support need to be aware that the balancing method, predictor variable selection and the virus type interact to affect the laboratory diagnosis of hepatitis virus infection with routine pathology laboratory variables in different ways depending on which combination is being studied. This awareness should lead to careful use of existing machine learning methods, thus improving the quality of laboratory diagnosis.

## Background

Both HBV and HCV are of global significance as leading causes of liver cancer (most common is HCC - Hepatocellular carcinoma). HCC ranks as approximately the seventh highest cancer worldwide, responsible for the third highest rate of cancer-related deaths, with 80% of HCC associated with chronic HBV or HCV [1, 2]. Pathology data collected via the health system provides a rich source of medical information on individual patients, and provides further benefits as a powerful aggregated database for complex modelling to investigate fundamental disease processes, as well as applied problems in laboratory diagnostics. With powerful computing and a variety of machine learning algorithms available and given the worldwide impact of HBV and HCV on human health, new diagnostic solutions through biomedical and quantitative science collaboration, opportunities to explore fundamental human health problems as well as systems-based issues, are possible.

Routine diagnostic pathology test results are an abundant data source for these sorts of complex analyses of aggregated patient physiological and biochemical responses to disease and infection events. Simple data mining techniques, like decision tree ensembles, have been applied previously to pathology laboratory data collected for the testing of patients suspected of Hepatitis B virus (HBV) or Hepatitis C virus (HCV) infection, detected by immunoassay for hepatitis B surface antigen (HBsAg) or serum anti-HCV antibody respectively [3]. Whether the prediction results of this previous study can be further optimised and improved are the focus to this current study.

Support vector machines (SVMs) have received considerable attention as a method of classification of large multi-dimensional data sets [4], and they will be the focus of this paper. The technique originated with research in statistical learning theory, with the basic idea to classify observations into two (or more) categories by finding the plane (in multiple dimensions) that best separates the multi-dimensional cloud of data points. SVMs are governed by choice of a small number of parameters, namely the kernel and the cost function, and so “state-of-the-art results can be achieved with relatively little effort” [5], p.214. SVMs have been applied to a variety of medical data including gene expression data [6]; and cancer imaging data [7].

For the models to be worthwhile, challenges within the data, such as imbalance between the number of individuals with positive versus negative immunoassay results, must be addressed. Classification of imbalanced data has attracted a large body of research over time, which has been reviewed recently [8]. These authors identified two basic strategies for dealing with imbalanced learning with a number of examples from the medical research literature: preprocessing techniques including resampling and feature selection [9], feature extraction [10] and cost-sensitive learning [11]; and classification algorithms including ensemble methods [12] and algorithmic classifier modifications [13]. SVMs have been used to determine the effect of a variety of balancing procedures on several publically available data sets [14]. Though SVMs have been found to be fairly robust to class imbalance [15], that study was highly structured in terms of predictors.

Simple downsizing has been found to be effective for a variety of data structures [16], and this study aims to identify whether simple downsizing is useful for highly imbalanced low-dimensional routine pathology data. Multiple downsizing has also been found to be effective in the context of high-dimensional data [10]: this study aims to identify whether a smaller lower-dimensional data set also benefits from multiple downsizing. Synthetic Minority Oversampling Technique (SMOTE) is the third method of data balancing investigated in this paper [17]. It has been used in a clinical context, for example to identify critically ill patients at risk of invasive infection [18].

A variety of methods for predictor variable selection within the SVM itself have also been proposed [19]. A typical SVM analysis includes all available predictor variables, but the presence of many predictors of low value can slow the calculations within the SVM. In this paper the effectiveness of random forests [20] as the feature selection method will be investigated.

The aims of the study were both to use SVMs to identify predictors for the enhanced laboratory diagnosis of hepatitis virus infection, and to identify the type of data balancing and feature selection that best assisted this enhanced classification of HBV/HCV negative or positive. Effective data balancing and feature selection to address the challenge of imbalanced data will enhance the timeliness and accuracy of models developed using SVMs on pathology data to predict laboratory outcomes, based on multiple biomarkers [21].

## Methods

### Data

The data set employed in this study originally comprised 18,625 individual cases (1 individual patient case per row) of hepatitis virus testing over a decade from 1997 to 2007. Data was provided by ACT Pathology, The Canberra Hospital (TCH), Australia and has also been analysed previously [3]. Patient identifiers were removed by TCH staff prior to data access, with only a laboratory ID numbers provided for the study. After data cleaning that included the removal of rows with missing values, 9170 rows of complete data were available for HBV tested patients (through HBsAg detection), and 7820 complete data rows available from HCV patients (via anti-HCV detection). Only the complete rows were used in the experiments described herein: approaches to missing data are described elsewhere [22]. The data access and analysis had Human Ethics Committee approval from ACT Health (protocol no. ETHLR.11.016) and The Australian National University (protocol no. 2012/349). A description of variables in the data set is shown in Table 1.

**Table 1:** Description of variables used in SVM analyses

Variable abbreviation	Description and definition	Measurement units
Response variables		
HBsAg	Hepatitis B Surface Antigen (marker of HBV infection)	Positive (1) or Negative (0)
HepC	Patient antibody to HCV, indicating contact with virus *(Both HBsAg and HepC detected by immunoassay)*	
Explanatory variables		
Age	Patient (case) Age	Years
Sex	Gender 1 = F, 2 = M	M or F
ALT	Alanine aminotransferase; an intracellular enzyme released after liver and other tissue cell damage	U/L
GGT	Gamma-glutamyl transpeptidase; an intracellular enzyme also relevant to liver damage	U/L
Hb	Haemoglobin	g/L
Hct	Haematocrit; formerly known as “packed cell volume”	%
Mch	Mean corpuscular haemoglobin	pg/RBC
MCHC	Mean corpuscular haemoglobin concentration	g/L
MCV	Mean corpuscular volume	f/L
Plt	Platelets; an agent in blood clotting	× 10^9^/L
WCC	White cell count	× 10^9^/L
RCC	Red cell count	× 10^12^/L
Crea	Creatinine; excreted by filtration through glomerulus and tubular section	μmol/L
K	Potassium; predominant intracellular cation whose plasma level is regulated by renal excretion	mmol/L
ALKP	Alkaline Phosphate; found in liver, bone, intestine and liver	U/L
ALB	Albumin; major component of plasma proteins	g/L
TBil	Total Bilirubin levels are reflective of the rate that the body recycles the red cells in the blood; bilirubin is a breakdown product of old, spent red blood cells.	μmol/L
Sodium	Sodium; predominant extracellular cation	mmol/L
Urea	Blood urea; often used to detect kidney related infections.	mmol/L
RDW	Red cell distribution width	%
Neut	Neutrophils; white blood cells, elevated by bacterial infection and early viral infection	× 10^9^/L
Lymph	Lymphocytes; white blood cells, elevated by viral infection and some cancers	× 10^9^/L
Mono	Monocytes; white blood cells, elevated by infection, inflammation, and some cancers	× 10^9^/L
Eos	Eosinophils; white blood cells, elevated by allergy and parasite infection	× 10^9^/L
Bas	Basophils; white blood cell, elevated in hypersensitivity reactions	× 10^9^/L

Serum HBsAg was classified as laboratory positive at ≥1.6 immunoassay units (IU) and HCV classified as positive at ≥1.0 IU. All other HBsAg and HCV results below this assay cut-off were classified as negative (M. de Souza, ACT Pathology, pers. comm.). No clinical notes were provided with the immunoassay data, so clinical infection status and symptoms were not known. Immunoassay results were obtained from the Architect HBsAg and anti-HCV platforms respectively (Abbott Australasia Pty Ltd. Diagnostics Division, North Ryde, NSW).

### Balancing

The high degree of imbalance in the data, with 172 out of 9170 (2%) rows HBV positive and 522 out of 7820 (7%) HCV positive, can perturb the optimal performance of support vector machines. We investigate simple downsizing, multiple downsizing, and SMOTE in this study.

Simple downsizing consisted in the current study of taking the large set of negative outcomes and randomly splitting it into subsets equal in size to the set of positive outcomes. Each subset of negative outcomes was put with the positive outcomes and the data analysis performed on that dataset. Simple downsizing for this investigation led to 52 sets of HBV-negative data and 13 sets of HCV-negative data of equal size, for analysis with the HBV-positive and HCV-positive data, respectively.

To implement multiple downsizing in the current study, a number of (we used 11) random selections of samples from the majority class were made and a classification rule was derived on each of the resulting balanced training sets (note that the minority class was the same in each training set). The 11 downsized classifiers were combined by majority voting; the predictions for observations in the testing set were obtained from each of the downsized classifiers and observations in the testing set were assigned to the predicted value with the larger number of votes. The multiple downsizing process was repeated ten times to allow for a measure of variability, similar to the measure of variability available across the 52 (for HBV) and 13 (for HCV) datasets to be analysed using simple downsizing.

The implementation of SMOTE was also tested for balancing the proportion of negative and positive outcomes in the dataset. SMOTE involves increasing the number of outcomes in the smaller group (in our case, positive test result) by synthesising data. At the same time, the larger group (in our case, negative test result) is decreased in size by sampling from it. The same number of SMOTE datasets (52 for HBV and 13 for HCV) as were available for simple downsizing were constructed using the DMwR library [23] in R [24].

### Scaling

Scaling methods [18] were considered for this study. However, these authors found that scaling and/or taking logs did not contribute significantly to accuracy in their experiment, so we will only report the results from raw data analysis here. Furthermore default data are scaled within the SVM to have zero mean and unit standard deviation, so externally imposed scaling is not necessary.

### Machine learning including predictor variable selection

We decided to investigate the usefulness of a two-step process involving constructing a random forest (RF), picking the top five variables in terms of mean decrease in the Gini index [20] and using only those five variables in the subsequent SVM. The same process was applied to both the downsized data sets and the SMOTE data sets.

We implemented the random forest (RF) analysis where required using the randomForest library [25]. We implemented the SVM analysis using the e1071 library [26]. Accuracy rates were measured by use of a 70% training, 30% testing split of the data [3, 27].

The order of work for downsizing and experimentation is shown in Table 2.

**Table 2:** Workflow for SVM analyses

HBV	
Extract 9170 individuals with HBV recorded of which 172 positive, 8998 negative	
Split data into training (70%) and testing (30%) with 120 positive and 6300 negative in each split	
Either	Downsize the training data into 52 sets of 120 positive plus 120 negative
Or	SMOTE the training data 400% oversampling and 100% under sampling leading to 52 sets of 3960 individuals with 1920 positive, 2040 negative
Or	Multiply downsize the training data into 11 sets of 120 positive and 120 negative
Then either	grow a random forest and pick the top five variables, apply SVM with the top five variables from the random forest
Or	proceed straight to SVM
HCV	
Extract 7820 individuals with HCV recorded with 533 positive, 7287 negative	
Split data into training (70%) and testing (30%) with 373 positive and 5100 negative in each split	
Either	Downsize the training data into 13 sets of 373 positive, 373 negative
Or	SMOTE the training data at 400% oversampling and 100% under sampling leading to 13 sets of 4797 individuals with 1492 positive, 1865 negative
Or	Multiply downsize the training data into 11 sets of 373 positive and 373 negative
Then either	grow a random forest and pick the top five variables, apply SVM with the top five variables from the random forest
Or	proceed straight to SVM

### Analysis of variance

The results are drawn together using an analysis of variance [28] to identify the amount of variation in three performance measures attributable to three factors. The performance measures were: sensitivity (the number positive and tested positive, divided by the number positive), precision (the number positive and tested positive, divided by the number tested positive) and F score (twice the product of precision and sensitivity, divided by the sum of precision and sensitivity). The three factors were data balancing, predictor variable selection and virus (HBV or HCV) analysed. The three data balancing methods were simple downsizing, multiple downsizing, and SMOTE. There were two techniques of predictor variable selection: none, and RF with the five most important variables fed back into an SVM. The interactions between pairs of the above factors were modelled for HBV and HCV separately (9170 observations for HBV and 7820 observations for HCV), to detect settings of one factor that cause the accuracy rates to behave differently depending on the setting of another interacting factor.

Finally, we use the best combination thus identified to find the assays that combine to best diagnose presence of HBV or HCV in the laboratory.

## Results

### Summary statistics

Summary statistics for patient demographics are shown in Table 3. The difference in mean age between HBV positive cases and controls was statistically significant (*t* = 4.33, df = 183.06, *p* < 0.0001) though the clinical difference of 5 years is not large and the ranges are similar (1 to 92 years for cases and 0 to 104 years for controls). The difference in mean age between HBV positive cases and controls was also statistically significant (*t* = 9.64, df = 679.58, *p* < 0.0001) though again, the clinical difference of 7 years is not large and the ranges are also similar (1 to 104 years for cases and 0 to 98 years for controls).

**Table 3:** Summary statistics for patient demographics

Variable	HBV positive (*n* = 172)	HBV negative (*n* = 8998)	*p*-value	HCV positive (*n* = 533)	HCV negative (*n* = 7287)	*p*-value
Sex	34% female	47% female	0.0008^a^	36% female	45% female	0.0001^a^
Age mean (s.d.)	40.5 (13.9)	45.2 (18.7)	0.0001^b^	40.6 (14.4)	47.1 (19.2)	<0.0001^b^

^a^Two-sample test of proportions, ^b^Two sample t test, *HBV* Hepatitis B Virus, *HCV* Hepatitis C virus

Means and confidence intervals of the three performance measures across the six different experimental settings are shown in Table 4. The spread of the performance measures is larger for HBV than HCV, which validates the decision to present the analyses for HBV and HCV separately. The means also show that SMOTE is an excellent method to counteract imbalance, except in terms of sensitivity to HCV.

**Table 4:** Sensitivity, precision and F scores by virus, balancing method and feature selection

HBV mean (95% CI)	SMOTE	SMOTE RF	Downsize	Downsize RF	MDS	MDS RF
Fscore	0.056 (0.054, 0.057)	0.052 (0.050, 0.053)	0.056 (0.054, 0.057)	0.052 (0.050, 0.053)	0.065 (0.061 0.068)	0.059 (0.055, 0.063)
Precision	0.034 (0.032, 0.036)	0.026 (0.025, 0.027)	0.029 (0.028, 0.030)	0.027 (0.026, 0.028)	0.034 (0.032, 0.036)	0.031 (0.029 0.032)
Sensitivity	0.625 (0.605, 0.645)	0.611 (0.587, 0.634)	0.625 (0.605, 0.645)	0.611 (0.587, 0.634)	0.246 (0.231, 0.260)	0.675 (0.654, 0.680)
HCV mean (95% CI)	SMOTE	SMOTE RF	Downsize	Downsize RF	MDS	MDS RF
Fscore	0.187 (0.179, 0.196)	0.200 (0.196, 0.202)	0.174 (0.170, 0.178)	0.208 (0.200, 0.215)	0.192 (0.190, 0.195)	0.225 (0.220, 0.229)
Precision	0.134 (0.128, 0.140)	0.117 (0.115, 0.119)	0.103 (0.100, 0.105)	0.124 (0.20, 0.129)	0.115 (0.113, 0.117)	0.138 (0.134, 0.141)
Sensitivity	0.311 (0.296, 0.326)	0.668 (0.654, 0.682)	0.567 (0.545, 0.590)	0.625 (0.600, 0.650)	0.589 (0.579, 0.598)	0.610 (0.596, 0.623)

*Downsize* simple downsizing, *Downsize RF* Simple downsizing with random forest variable selection, *MDS* Multiple downsizing, *MDS RF* MDS with random forest variable selection, *SMOTE* Synthetic Minority Oversampling Technique, *SMOTE RF SMOTE* with random forest variable selection, *HBV* Hepatitis B virus, *HCV* Hepatitis C virus

### Effect of balancing method

The effect of balancing method on the three performance measures is displayed in Tables 5 and 6. For HBV (Table 5), method has a significant effect on precision. Follow-up analyses when RF is used show the contributors to the significant method effect are MDS-downsize (diff = 0.0038, *p* = 0.0001) and SMOTE-MDS (diff = −0.0044, *p* < 0.0001). When RF is not used, the contributors to the significant method effect are MDS-downsize (diff = 0.0049, *p* = 0.0187) and SMOTE-downsize (diff = 0.0049, *p* < 0.0001).

**Table 5:** Analysis of variance of F score, precision and sensitivity by balancing method and feature selection for HBV

Precision source	SS	df	MS	F	p
Method	0.0004	2	0.0002	13.088	0.000 (^a^)
Pre-processing	0.0013	1	0.0013	80.504	0.000 (^a^)
Method.Pre-processing	0.0004	2	0.0002	12.222	0.000 (^a^)
Sensitivity Source	SS	df	MS	F	p
Method	1.6151	2	0.8075	159.98	0.000 (^a^)
Pre-processing	1.9877	1	1.9877	393.78	0.000 (^a^)
Method.Pre-processing	2.8062	2	1.4031	277.97	0.000 (^a^)
F score Source	SS	df	MS	F	p
Method	0.0011	2	0.0006	10.838	0.000 (^a^)
Pre-processing	0.0025	1	0.0025	47.154	0.000 (^a^)
Method.Pre-processing	0.0003	2	0.0002	3.007	0.052

(^a^) = Significant at 0.0025 level with adjustment for multiple testing. Method = simple downsizing, multiple downsizing or SMOTE. Pre-processing = random forest variable selection or not. Method.Pre-processing = the interaction between Pre-processing and Method

Similarly, for HBV, method has a significant effect of sensitivity. Follow-up analyses when RF is used show the contributor to the significant method effect is SMOTE-downsize (diff = 0.0625, *p* = 0.0002). When RF is not used, all three pairs of method are contributors to the significant method effect.

Finally, for HBV method has a significant effect on F score. Follow-up analyses show the contributors to the significant effect are MDS-downsize (diff = 0.0082, *p* = 0.0001) and SMOTE-MDS (diff = −0.0066, *p* < 0.0001).

For HCV, method has a significant effect on precision. Follow-up analyses when RF is used show the contributors to the significant method effect are MDS-downsize (diff = 0.134, *p* < 0.0001), SMOTE-downsize (diff = −0.0076, *p* = 0.0038) and SMOTE-MDS (diff = −0.0209, *p* < 0.0001). When RF is not used, all three pairs of method are contributors to the significant method effect.

Similarly, for HCV (Table 6), method also has a significant effect on sensitivity. Follow-up analyses when RF is used show the contributors to the significant method effect are SMOTE-downsize (diff = 0.0428, *p* = 0.0029) and SMOTE-MDS (diff = 0.0582, *p* = 0.0002). When RF is not used, the contributors to the significant method effect are SMOTE-downsize (diff = −0.2563, *p* < 0.0001) and SMOTE-MDS (diff = −0.2777, *p* < 0.0001).

**Table 6:** Analysis of variance of F score, precision and sensitivity by balancing method and feature selection for HCV

Precision source	SS	df	MS	F	p
Method	0.0025	2	0.0013	32.843	0.000 (^a^)
Pre-processing	0.0011	1	0.0011	28.713	0.000 (^a^)
Method.Pre-processing	0.0064	2	0.0032	84.402	0.000 (^a^)
Sensitivity Source	SS	df	MS	F	p
Method	0.0194	2	0.0970	114.86	0.000 (^a^)
Pre-processing	0.4375	1	0.4375	518.21	0.000 (^a^)
Method.Pre-processing	0.4162	2	0.2081	246.46	0.000 (^a^)
F score Source	SS	df	MS	F	p
Method	0.0041	2	0.0021	25.546	0.000 (^a^)
Pre-processing	0.0114	1	0.0114	141.771	0.000 (^a^)
Method.Pre-processing	0.0019	2	0.0010	11.844	0.000 (^a^)

(^a^) = Significant at 0.0025 level with adjustment for multiple testing. Method = simple downsizing, multiple downsizing or SMOTE. Pre-processing = random forest variable selection or not. Method. Pre-processing = the interaction between Pre-processing and Method

Finally, for HCV, method has a significant effect on F-score. Follow-up analyses when RF is used show all three pairs of method are contributors to the significant method effect. When RF is not used, the contributors to the significant method effect are MDS-downsize (diff = 0.0184, *p* = 0.0001) and SMOTE-downsize (diff = 0.0133, *p* = 0.0027).

### Effect of feature selection via random forest

The effect of feature selection is also shown in Tables 5, 6. For HBV (Table 5), feature selection has a significant effect on precision. When downsizing is used, the effect is significant (diff = −0.0024, *p* = 0.0002). When SMOTE is used the effect is also significant (diff = −0.0077, *p* < 0.0001) but the effect when multiple downsizing is used is not significant.

Similarly for HBV, feature selection has a significant effect on sensitivity. SMOTE is the only method contributing to this significance (diff = 0.4290, *p* < 0.0001).

Finally, for HBV, feature selection has a significant effect on F-score (diff = −0.0066, *p* < 0.0001), and the difference is not statistically significant according to which method is employed.

For HCV (Table 6), feature selection has a significant effect on precision, sensitivity and F score. All three methods contribute significantly to the effect for all three performance measures.

**Fig. 1:**
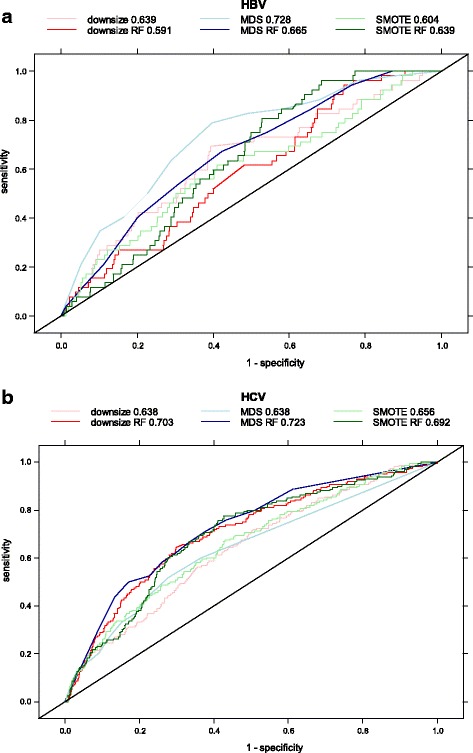
Receiver-operator characteristic (ROC) curves for **a** HBV and **b** HCV summarising the six models under consideration for improving prediction for imbalanced data. The models are: Downsize = simple downsizing; Downsize RF = simple downsizing with random forest variable selection; MDS = multiple downsizing; MDS RF = multiple downsizing with random forest variable selection; SMOTE = Synthetic Minority Oversampling; SMOTE RF = Synthetic Minority Oversampling with random forest variable selection

To test the ability of each approach to distinguish positive from negative test results, the area under the receiver operating characteristic curve (AUC) was calculated (Fig. 1). For HBV (Fig. 1a), the AUC values lie between 0.591 and 0.728 which indicate all approaches are good but not excellent (defined by AUC > 0.8). For downsizing and MDS, addition of predictor variable selection by RF improved the AUC, but the reverse outcome was found for SMOTE. The method that produced the greatest AUC was MDS. For HCV (Fig. 1b), the AUC values lie between 0.638 and 0.723, a tighter range than for HBV. These AUC values also indicate that all methods were good, but not excellent as defined above. With no variable selection, SMOTE had the largest AUC, whereas with variable selection, MDS had the largest AUC. For any one approach, addition of variable selection by RF improved the AUC.

### Key predictor variables and clinical impact

The clinical question of which variables are the most important for predicting positive immunoassay results for HBV and HCV is scattered due to the large number of datasets that arise from downsizing, and the multiple datasets generated under SMOTE that provided the replication needed for the analysis of variance. So the clinical question is addressed on the entire dataset in the following manner. For HBV SMOTE was applied to the entire dataset with an over-sampling fraction of 400% and an under sampling fraction of 100%, after which a RF was grown to establish the three most important variables that were then fitted an SVM. For HCV an identical SMOTE strategy was applied, but with an oversampling fraction of 100% and an under-sampling fraction of 100%. The three most important variables were identified by RF and then fitted to a SVM using ten-fold cross-validation, a cost parameter optimised over the range {10, 100} and a scaling parameter, gamma, optimised over the range {0.001, 0.1}.

**Fig. 2:**
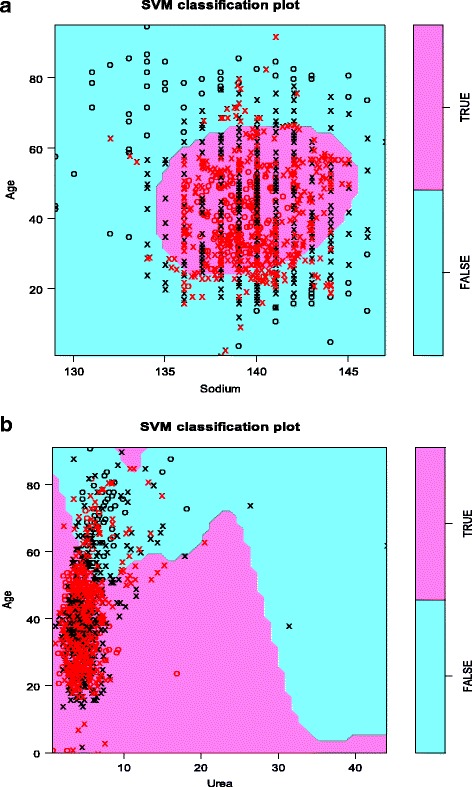
**a** Hepatitis B virus (HBV) and **b** Hepatitis C virus (HCV) SVM plots post SMOTE to overcome HBV/HCV immunoassay class imbalance, and random forest to identify the top three predictors of HBV or HCV positive/negative immunoassay class. For HBV and HCV SVM visualisation, the SVM was sliced at ALT equal to 35 IU/L

For the entire HBV data set, the three most important variables were ALT, Age and Sodium in decreasing order. For HCV they were Age, ALT and Urea. By fitting an SVM using three variables, it is possible to visualise the results of the predictions (Fig. 2).

For HBV, Age, ALT and Sodium were the top three predictors. Figure 2a shows values of Sodium and Age on the x and y axes respectively, for a constant serum ALT concentration of 35 IU/L. Black data points are used in the construction of the SVM, red ones are not. HBV positive cases are identified by crosses, and controls by circles. The central coloured shape shows that HBV infection will be predicted (HBV = TRUE) when Age is between 25 and 65 years, and Sodium between 135 and 145 mmol/L. The ten-fold cross-validation accuracy of this model is 64%, with individual accuracies in the folds ranging from 55 to 69%. For HCV (Fig. 2b) at the same constant ALT concentration, HCV infection will generally be predicted when Age is below 60 years and Urea is below 40 mmol/L, and when the combination of the two is below around 60 (due to the sloping boundary between the two coloured areas). The ten-fold cross-validation accuracy of this model is 71%, with individual accuracies in the folds ranging from 68 to 76%.

To summarise, the enhanced laboratory prediction of HBV infection (as detected by HBsAg immunoassay), after balancing to account for the class imbalance for HBsAg negative versus positive results via SMOTE, showed that balancing method, feature selection and their interaction were statistically significant. Based on raw data, the key predictors were Age, serum ALT and sodium. Also, the accuracy rate of SVM was calculated as approximately 64% for SMOTE data, and if high sensitivity is a priority, inclusion of variable selection with a random forest prior SVM is advantageous. For HCV laboratory diagnosis, balancing method, feature selection and interaction were statistically significant, and based on raw data the key predictors were Age, serum ALT and Urea for SMOTE data. An accuracy rate of SVM of approximately 71% for SMOTE data was achieved, and as for HBV, if high sensitivity is a priority include variable selection with SMOTE.

## Discussion

### Specific contribution

The contribution of this paper to the understanding of optimisation for SVM modelling of routine pathology blood test results, associated with HBV or HCV positive/negative results as response categories, will help achieve such diagnostic laboratory alternatives where resources are limited or absent. Utilising routine results as presented here, such algorithms allow the early detection of HBV or HCV infection; hepatitis immunoassay is a special test ordered as a results of other investigations, and as such is not routinely requested, potentially missing cases of infection.

### Comparison to existing literature

The interrogation of routine pathology data associated with positive/negative HBV and HCV immunoassay data has been performed previously through single decision tree and tree ensemble analysis to examine the effect of data balancing, feature selection, and other adjustments to enhance HBV and HCV immunoassay prediction [3].

With significant data imbalance and the need for feature selection, the high rates of prediction accuracy, sensitivity and specificity achievable in a Chinese cohort of 518 chronic HBV patients, compared to 303 HBV and HCV negative controls [21], were not matched here. The higher degree of balance between HBsAg positive and negative test results clearly contributed to the higher sensitivity and specificity values observed in that paper.

Comparable AUC values have been achieved by a similar study of pathology data [29], focusing on one or two classification methods but including longitudinal data which was not available for the current study.

### Implications for practice

From the biomedical and diagnostic perspective, this study emphasised the importance of age and alanine aminotransferase (ALT) as of highest importance to accurate hepatitis prediction, followed by Sodium for HBV and Urea for HCV. Key variables detected by the current study were scattered because of the multiple datasets utilised in these analyses, but some predictor variables emerge of potential diagnostic importance. Of note among predictor variables was the strong association of age to the positive detection of HBsAg, indicating significantly higher HBV infection prevalence in patients between 20 and 60 years old. This suggests that the higher prevalence of risk-taking behaviour (e.g. intravenous drug use) for young adults, and into middle age, is a key predictor for HBV infection, an observation reinforced by epidemiological studies of drug, alcohol and other high risk behaviours across the general population in Australia [30].

Given the extensive range of biochemical, cellular and physiological data associated with HBV or HCV immunoassay, via simultaneously collected routine pathology results, support vector machines and the SMOTE technique offer the opportunity to use an entire data set to enhance the laboratory prediction of infectious diseases, particularly where a large disparity exists between the number of negative and positive test results.

### Limitations

Several considerations particularly arise in regards to the limitations of this paper. First, only two datasets have been examined, pertaining to HBV and HCV respectively. The best methods identified in this paper may not apply to laboratory diagnosis of other infections, or the same infections in other settings. Second, a limited set of features (30 biomarkers) was available for analysis and these models are naturally limited by the features available.

Third, the balance in the datasets examined is extreme, with little more than 2% of positive test results. This degree of imbalance is common in the diagnostic pathology context. One-class classification can be used in such situations however researchers must be sure that the method matches the research question [31]. The sparse literature on the use of one-class classification in medical research was noted in 2010 [32], and has not expanded to a very great extent since then. The availability of open source software is also a limitation to the implementation of such methods in laboratory settings.

Finally, the balancing methods that were examined were simple downsizing, multiple downsizing and SMOTE; and the feature selection method that was examined was the random forest.These were selected for their durability in the literature to date and their transparency for clinical use. Many other algorithms exist and are in development: future work should investigate the best of these new procedures.

## Conclusion

This study examined the effect of balancing and feature selection on the performance of SVMs used to predict HBV or HCV infection status, as detected by specific immunoassay. SMOTE has been shown to enhance prediction accuracy considerably, compared to decision trees, simple downsizing and multiple downsizing methods. The effect of predictor variable selection has been shown to be of smaller practical significance even though statistical significance is achieved.

Better understanding of the behaviour of data mining techniques will lead to enhanced laboratory prediction of infections such as HBV and HCV once data mining algorithms are embedded in the analysis of routine pathology data.
